# Probable Vogt–Koyanagi–Harada disease with initial unilateral ocular manifestation in a hepatitis C carrier

**DOI:** 10.1007/s12348-012-0082-x

**Published:** 2012-05-16

**Authors:** Nikki Y. Far, David T. L. Liu

**Affiliations:** 1 Department of Ophthalmology and Visual Sciences, The Chinese University of Hong Kong, Hong Kong, People’s Republic of China; 2 Department of Ophthalmology and Visual Sciences, University Eye Center, 3/F, Hong Kong Eye Hospital, Hong Kong, People’s Republic of China

## Introduction

Vogt–Koyanagi–Harada (VKH) disease is a systemic disorder with typical bilateral granulomatous panuveitis and variable degrees of systemic features, including meningismus, vitiligo, poliosis, and dysacusis. The exact pathogenesis remains unknown, and a theory of autoimmune response against certain antigenic component of melanocytes has been suggested [1]. Diagnosis is mainly based on clinical findings. Revised diagnostic criteria for VKH disease stressed the importance of the presence of bilateral uveitis [2]. Most patients do present with bilateral ocular involvement, where onset in the second eye might show 2 to 3 weeks of delay than the first eye. However, several cases of unilateral disease have also been reported after long-term follow-up [3]. Nowadays, the mainstay of treatment would be high dose systemic corticosteroid, although steroid-sparing agents should be considered in patients unable to tolerate steroid or in those who have developed steroid dependence [4].

## Case report

**Fig. 1:**
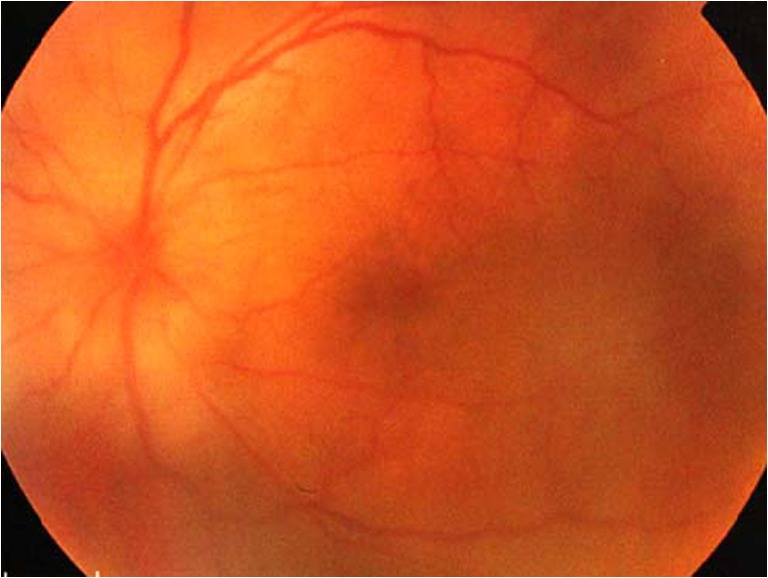
Left fundus showed multiple pockets of serosanguineous detachment, underlying choroidal inflammatory foci, and a disc swelling on initial presentation

**Fig. 2:**
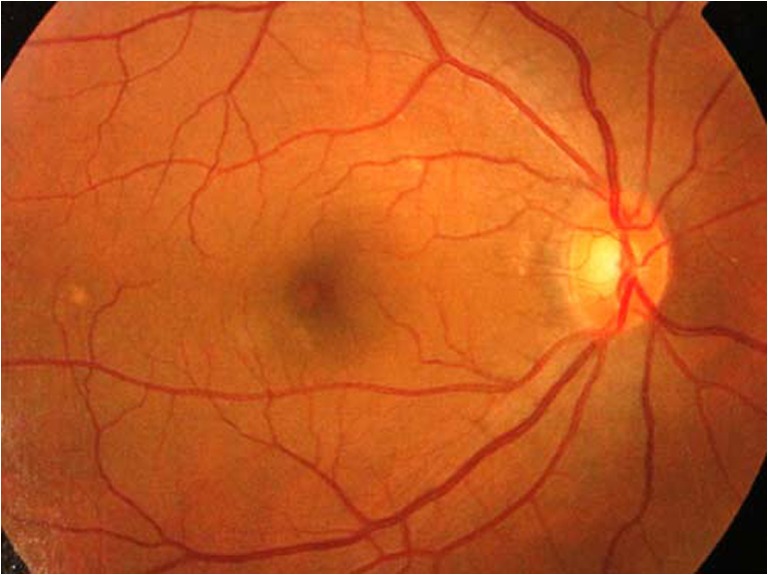
Normal right fundus on initial presentation

A 49-year-old gentleman first presented to our ophthalmology clinic with 1 month history of left eye pain, redness, and photophobia in August 2006. He was previously an intravenous drug user. His ocular symptoms did not respond to topical antibiotics prescribed by general practitioners. No rheumatological, neurological, auricular, or cutaneous features were noted. Neither did he have any preceding history of ocular trauma or surgery. Slit lamp examination of the left eye revealed grade 2 anterior chamber and vitreous inflammation, according to the Standardization of Uveitis Nomenclature classification. Fundoscopy of the left eye showed multiple pockets of serosanguineous detachment at the posterior pole and perivascular arcade areas with underlying choroidal inflammatory foci evident, in keeping with diffuse choroiditis (Fig. 1). A disc swelling and early peripapillary nerve fiber layer edema were noted as well (Fig. 1). Findings in the right eye were within normal limits (Fig. 2). Serum anti-HCV was positive. Liver function was normal. Serum antibody titres for HBV, HIV, and *Treponema pallidum* were negative. Mantoux test was unremarkable and both Tspot/Quantiferon tests were negative. Anti-nuclear antibody was elevated to 80 (speckled pattern, normal <40).

Oral prednisolone (1 mg/kg/day) and topical pred forte eye drop were started after discussion with hepatologists. Both were gradually tapered over a period of 13 months. Sunset-glow fundus was noted in the left eye 5 months after onset. Right eye examination findings were within normal limits at that juncture.

Initial HCV RNA titre was 3.45 × 10^5^ IU/ml. A 24-week course of antiviral therapy (Ribavirin and peginterferon alfa-2A) was given between December 2007 and June 2008. Posttreatment HCV RNA titre was undetectable, indicating eradication.

Shortly after the antiviral therapy, the patient presented with a decrease in vision in the right eye in July 2008 (i.e., 2 years after the first onset). Fundoscopic exam revealed right eye diffuse choroiditis and exudative retinal detachment. The left eye showed a similar sunset-glow fundus as in the previous review without any signs of active inflammation. Fluorescein angiography showed multifocal of pinpoint leakage over right eye. A cerebrospinal fluid study found no pleocytosis or presence of malignant cells. A second course of oral prednisolone (1 mg/kg/day) and topical steroid was prescribed and tapered off over 5 months. Unfortunately, a repeated HCV RNA titre during that period was 8.27 × 10^5^ IU/ml, indicating a relapse of hepatitis C. In view of the relapsing VKH disease, hepatologists decided to withhold any further antiviral therapy and would continue regular monitoring of liver enzymes level and ultrasonography.

**Fig. 3:**
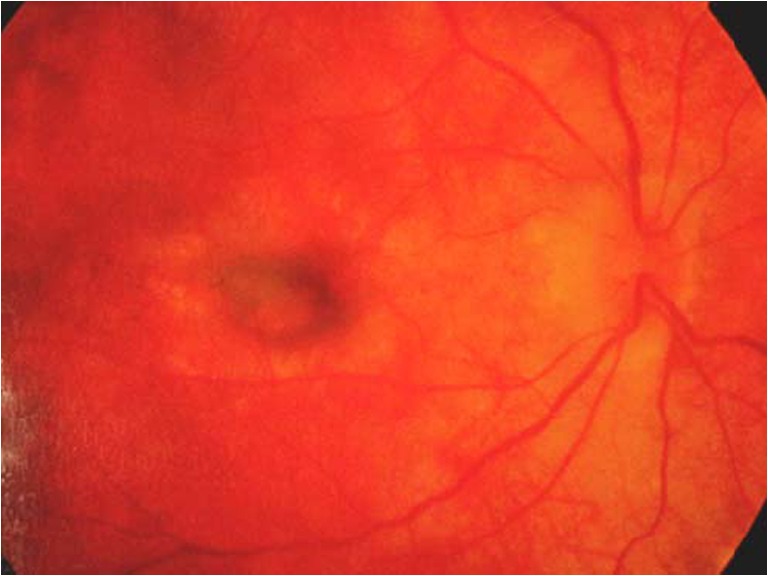
Right fundus with features of choroidal neovascularization

The patient developed a relapse by presenting with panuveitis in the form of vitritis and anterior uveitis in February 2009. The result of a left fundoscopic exam found sunset-glow changes but no active inflammation. He was resumed on oral prednisolone (1 mg/kg/day) and topical steroid, with a prolonged treatment course lasting 30 months. Difficult tapering with frequent flare-ups of the right eye uveitis was noted. Furthermore, the right eye developed a complication by the formation of choroidal neovascularization (Fig. 3) for which photodynamic therapy was given. Systemic steroid was finally stopped in September 2011.

The patient remained stable without active ocular inflammation while he was put on topical pred forte. His latest visual acuity was 12/200 in the right eye and 20/40 in the left eye. No systemic features developed all along.

## Discussion

The revised diagnostic criteria for VKH disease includes: (1) no history of penetrating ocular trauma or surgery preceding the onset of uveitis; (2) no evidence suggestive of other ocular disease; (3) bilateral ocular involvement with diffuse choroiditis at early stage or ocular depigmentation at late stage of the disease; (4) neurological or auditory manifestations, e.g., meningismus and tinnitus; and (5) integumentory findings, e.g., alopecia, poliosis, and vitiligo [2]. Our patient fulfilled the criteria for probable VKH disease with isolated ocular involvement. No systemic manifestations were identified even after 5 years of follow-up. It was interesting to note that our patient had 2 years of delay in his second eye with demonstrable signs of VKH disease, whereas majority of patients only showed a few weeks of delay. Both eyes had sunset-glow changes at the end of the follow-up, which was consistent with the diagnosis of VKH disease.

Concomitant hepatitis C and steroid dependance raised challenges for management. In general, for patients who are intolerable to systemic steroid or develop steroid dependance, immunomodulatory agents, such as methotrexate, cyclosporine, and azathioprine, could be considered. However, with the presence of hepatitis C, the choice of immunosuppressants became markedly limited in view of potential flare-up of hepatitis and hepatotoxicity. Our patient showed a fluctuating clinical course despite prolonged use of high dose prednisolone. At our latest review, his disease activity had been controlled with topical steroid. In case of further flare-ups, sub-tenon steroid injection could also be considered for his isolated ocular disease. There were several case reports of VKH-like disease after administration of interferon alpha in patients with hepatitis C [5–7]. The exact mechanism was unknown but a T cell-mediated immune response was suspected. It would be important to closely monitor his ocular signs and symptoms in case of further treatment with interferon alpha in the future.

The clinical and laboratory findings of the patient were characteristic of a probable VKH disease except for the initial unilateral involvement for 2 years. Steroid dependence and concomitant hepatitis C raised challenges to clinicians in management. Alternatives to systemic immunosuppressants could be considered. Despite being a rare variant, it is important for ophthalmologists to recognize a unilateral presentation of VKH disease.
